# Aloe-emodin plus TIENAM ameliorate cecal ligation and puncture-induced sepsis in mice by attenuating inflammation and modulating microbiota

**DOI:** 10.3389/fmicb.2024.1491169

**Published:** 2024-12-12

**Authors:** Jingqian Su, Xiaohui Deng, Shan Hu, Xinrui Lin, Lian Xie, Hui Ye, Congfan Lin, Fen Zhou, Shun Wu, Liling Zheng

**Affiliations:** ^1^Fujian Key Laboratory of Innate Immune Biology, Biomedical Research Center of South China, College of Life Science, Fujian Normal University, Fuzhou, Fujian, China; ^2^First Hospital of Quanzhou Affiliated to Fujian Medical University, Quanzhou, Fujian, China

**Keywords:** aloe-emodin, TIENAM, sepsis, anti-inflammatory, combination therapy, peritoneal cavity microbiota

## Abstract

Despite the high sepsis-associated mortality, effective and specific treatments remain limited. Using conventional antibiotics as TIENAM (imipenem and cilastatin sodium for injection, TIE) is challenging due to increasing bacterial resistance, diminishing their efficacy and leading to adverse effects. We previously found that aloe-emodin (AE) exerts therapeutic effects on sepsis by reducing systemic inflammation and regulating the gut microbiota. Here, we investigated whether administering AE and TIE post-sepsis onset, using a cecal ligation and puncture (CLP)-induced sepsis model, extends survival and improves physiological functions. Survival rates, inflammatory cytokines, tissue damage, immune cell populations, ascitic fluid microbiota, and key signaling pathways were assessed. Combining AE and TIE significantly enhanced survival rates, and reduced inflammation and bacterial load in septic mice, indicating potent antimicrobial properties. Moreover, substantial improvements in survival rates of AE + TIE-treated mice (10% to 60%) within 168 h were observed relative to the CLP group. This combination therapy also effectively modulated inflammatory marker (interleukin [IL]-6, IL-1β, and tumor necrosis factor [TNF]-α) levels and immune cell counts by decreasing those of B, NK, and TNFR2+ T_reg_ cells, while increasing that of CD8+ T cells; alleviated tissue damage; reduced bacterial load in the peritoneal cavity; and suppressed the NF-κB signaling pathway. We also observed a significantly altered peritoneal cavity microbiota composition post-treatment, characterized by reduced pathogenic bacteria (*Bacteroides*) abundance. Our findings underscore the potential of AE + TIE in treating sepsis, and encourage further research and possible clinical implementations to surmount the limitations of TIE and amplify the therapeutic potential of AE.

## 1 Introduction

Sepsis, a severe medical condition initiated by infection, leads to organ dysfunction arising from a disruption in the balance between inflammation and immune suppression. Globally, it impacts around 18 million people annually and carries a mortality rate ranging from 28% to 50% (Singer et al., 2016; Rudd et al., 2020). Despite its clinical importance, effective specific therapies are scarce (Lindell and Meyer, 2024). Recent advancements in machine learning and artificial intelligence have shown significant promise in improving sepsis detection and management (Agnello et al., 2024). These technologies leverage vast clinical data to identify patterns and predict patient outcomes, enabling earlier intervention. Machine learning algorithms, such as random forests and neural networks, have been used to analyze electronic health records and vital sign data, enhancing diagnostic accuracy (Li et al., 2024). Traditional approaches have primarily aimed at controlling inflammation. Traditionally, the treatment of sepsis has relied on the use of antibiotics (Asner et al., 2021; Sterling et al., 2015), fluid resuscitation (Tseng et al., 2020), and supportive care (Perner et al., 2017). Early antibiotic therapy is considered to be an important factor in improving the prognosis of patients with sepsis, while fluid resuscitation is a key measure in maintaining hemodynamic stability. However, emerging evidence underscores the necessity to simultaneously address both pro-inflammatory and anti-inflammatory responses for effective sepsis management (Huang et al., 2019). Variabilities in the outcomes of anti-inflammatory treatments underscore the potential benefits of restorative immunotherapy, given the disease's complexity (Giamarellos-Bourboulis et al., 2024).

Research into sepsis utilizes models generated through *Escherichia coli* infusion, lipopolysaccharide (LPS) infusion, and cecal ligation and puncture (CLP). These models introduce a combination of intestinal bacteria, fungi, and metabolites into the peritoneal cavity, closely replicating human sepsis conditions (Korneev, 2019; Fernández et al., 2021). Such models often result in widespread infection and inflammation, reflecting an intricate link with intestinal microbiota imbalance, the dynamics of which remain poorly understood (Kullberg et al., 2021).

Present treatment strategies for sepsis predominantly rely on antibiotics, which introduce risks such as resistance development and microbiota disruption, complicating the long-term management of the condition (Cook and Wright, 2022; Aghai, 2020). TIENAM (imipenem and cilastatin sodium for injection, TIE), marketed as Primaxin, is a combination of imipenem, a broad-spectrum thienamycin-class antibiotic, and cilastatin sodium, which inhibits renal enzyme dehydropeptidase I (Motsch et al., 2020). Although these antibiotics are recognized for their broad antimicrobial capabilities, concerns regarding resistance development and potential organ damage from extended use continue to persist (Qin et al., 2022).

Aloin (CAS: 1415-73-2) and aloe-emodin (AE; CAS: 481-72-1), derived from various *Aloe* species, are natural anthraquinone compounds (Xiao et al., 2022). Within the intestinal tract, aloin is enzymatically converted into AE, which then stimulates the intestinal walls, boosts peristalsis, and facilitates the removal of waste (Yang et al., 2021; Friedman et al., 2020). Aloin and aloe-emodin are natural anthraquinone compounds derived from various species of Aloe (Xiao et al., 2022). Although both compounds originate from the same plant family, they exhibit notable differences in chemical structure, biological activity, and physiological effects. Aloin, primarily found in the latex of *Aloe* leaves, is well-known for its laxative properties by stimulating intestinal peristalsis (Yang et al., 2021). Aloe-emodin is formed when Aloin undergoes enzymatic conversion in the intestine. Aloe-emodin, an anthraquinone derivative, demonstrates a broader range of biological activities, including anti-inflammatory, antimicrobial, and potential anticancer effects (Friedman et al., 2020). While Aloin is primarily recognized for its role as a laxative, aloe-emodin has attracted attention for its therapeutic potential beyond digestive health. Recent work by Gao et al. (2022) has demonstrated protective effects of AE against LPS-induced inflammation in a mouse model. Our research group previously found that AE administered at a dosage of 10 mg/kg/day significantly enhanced survival rates and reduced infection in mice with sepsis (Su et al., 2023a).

Nevertheless, the crucial scientific and clinical tasks of boosting efficacy, enhancing survival rates, managing infections, curbing inflammation, and averting bacterial drug resistance are of utmost importance.

In our prior research, we demonstrated that combining Aloin or ulinastatin with TIE effectively improves outcomes in CLP- induced sepsis mice by reducing inflammation and modulating immune responses. Building on these findings, this research aims to introduce a novel therapeutic strategy by combining AE with TIE (Su et al., 2024d,f).

While AE has shown promise in reducing systemic inflammation and regulating gut microbiota, its individual therapeutic effect is insufficient. Similarly, TIE is a powerful antibiotic, but its use is increasingly challenged by the rising bacterial resistance and associated adverse effects. Combining AE with TIE addresses these limitations, offering a more comprehensive treatment approach. This strategy not only expands the therapeutic options for sepsis but also improves the overall management of the condition, potentially improving patient outcomes and informing future therapeutic strategies.

## 2 Materials and methods

### 2.1 Reagents and chemicals

AE was procured from Shanghai Yuanye Bio-Technology Co., Ltd. (Shanghai, China). TIE was sourced from Merck Sharp & Dohme (China). Additionally, Reverse Transcription Green qPCR SuperMix and bicinchoninic acid kits for protein concentration determination were procured from TransGen (Beijing, China). Enzyme-linked immunosorbent assay (ELISA) kits from R&D Systems (Minneapolis, MN, USA) were also used. Table 1 comprehensively details the antibodies used in this study, along with information on their providers.

**Table 1:** Antibodies used in the study.

**Antibody**	**Manufacturer**	**Identifier**
CD45 BUV395-A	Becton, Dickinson and Company	Cat#564279
CD11b BB515-A	Becton, Dickinson and Company	Cat#557397
BV421 F4/80-A	Becton, Dickinson and Company	Cat#564088
CD3 APC-Alexa 700-A	Becton, Dickinson and Company	Cat#561388
CD8 BUV805-A	Becton, Dickinson and Company	Cat#612898
CD25 BB700-A	Becton, Dickinson and Company	Cat#567482
CD20 PE-BYG568-A	Becton, Dickinson and Company	Cat#152106
NK1-1 PE-CF594-A	Becton, Dickinson and Company	Cat#562864
CD69 BV711-A	Becton, Dickinson and Company	Cat#740664
Fixable Viability Stain 780	Becton, Dickinson and Company	Cat#565388
NF-κB	ABclonal	A19653
p-NF-κB	ABclonal	AP0475
GAPDH	ABclonal	A19056
β-actin	Servucebio	GB15001-100
IKBα	Cell Signaling Technology	Cat#48125
p-IKBα	Cell Signaling Technology	Cat#2859T

### 2.2 Animals

Pathogen-free C57BL/6 mice, aged 8–10 weeks, were acquired from Shanghai SLAC Laboratory Animal Co., Ltd. (Shanghai, China), equally divided by sex, and housed at the Experimental Animal Center of Fujian Normal University. Mice were maintained under a controlled environment with temperature set between 23°C and 25°C, humidity levels between 40% and 60%, and a 12 h light/dark cycle. Male mice weighing 20–22 g and female mice weighing 18–20 g were provided *ad libitum* access to food and water, and were randomly assigned to various experimental groups. A total of 160 mice were used in this study, with an equal distribution of females (*n* = 80) and males (*n* = 80). These mice were randomly assigned to 8 groups, each consisting of 20 mice: sham, sham + AE, sham + TIE, sham + AE + TIE, CLP (sepsis model), CLP + AE, CLP + TIE, and CLP + AE + TIE. The treatments with AE (10 mg/kg/day), TIE (50 mg/kg/day), and the combination of AE + TIE were administered 1 h after sepsis induction.

### 2.3 CLP model of sepsis

The CLP procedure used to induce sepsis followed a well-documented protocol (Alverdy et al., 2020; Su et al., 2023b). Mice were anesthetized via intraperitoneal injection of 0.3% pentobarbital sodium. Subsequently, mice were placed on the surgical platform, and the lower left quadrant of their abdomen was shaved using an electric razor. The area was initially sterilized using a betadine solution, followed by 75% alcohol. An incision was made along the length of the specific peritoneal region, followed by a 1 cm cut through the outer layers to expose the cecum. Approximately two thirds of the cecum were tightly ligated near its base using a sterile 4.0 surgical thread. A small hole was then made in this area using a clean 5 mL syringe needle, allowing a small amount of fecal matter to extrude. After ligation and perforation, the cecum was gently placed back into the peritoneal cavity, and the incisions in the muscle and skin were sutured using a sterile 4.0 suture. To replenish fluids, a subcutaneous injection of 1 mL of pre-warmed 0.9% saline solution was administered on the back. Following surgery, mice were placed on a heated mat to ensure full recovery from anesthesia. In CLP model, the locus of sepsis is centered on the peritoneal cavity, specifically targeting the cecum as the primary site of infection. This site allows for controlled release of fecal matter into the peritoneal space, mimicking the polymicrobial nature of human intra-abdominal sepsis. The sham control group underwent only a laparotomy, without cecal ligation or puncture.

### 2.4 Study design

Mice were allocated into five experimental groups: sham (control), CLP (sepsis model), AE treatment (10 mg/kg/day), TIE treatment (50 mg/kg/day), and a combination of AE and TIE treatments. Treatments with AE and TIE commenced 1 h post-sepsis induction. Survival rates were observed at 6-h intervals post-administration. The severity of sepsis was assessed using the murine sepsis score (MSS) (Shrum et al., 2014). This scoring system assesses factors such as body temperature, activity levels, appearance, and breathing patterns. Each parameter is scored on a scale from 0 to 4, with higher scores indicating more severe symptoms of sepsis. The overall MSS is the sum of the individual scores, providing a quantitative measure of sepsis progression in the animals. The mice were monitored for signs consistent with systemic inflammatory response syndrome (SIRS), including elevated heart rate, respiratory rate, temperature changes, and leukocytosis, following the established clinical criteria adapted for animal models (Serafim et al., 2018). Following anesthesia with sodium pentobarbital, the systematic collection of blood, fecal matter, ascitic fluid, and tissue samples was performed according to established protocols (Wang P. et al., 2021).

### 2.5 Quantitative reverse transcription PCR analysis

Total RNA was isolated using TRIzol reagent (Takara, Tokyo, Japan), following the RT-qPCR methodologies outlined in a previous study (Wang Y. et al., 2021). mRNA levels were quantified utilizing specific primers detailed in Table 2 and were normalized against GAPDH as a reference gene. The expression changes relative to the expression in the phosphate-buffered saline (PBS) control were calculated employing the ΔΔCt method. This analysis was repeated thrice for each gene to confirm the precision and consistency of the data.

**Table 2:** Sequence of the primers.

**Name**	**Sequence (5^′^– 3^′^)**
*IL-6*	F: TACCACTTCACAAGTCGGAGGC
	R: CTGCAAGTGCATCATCGTTGTTC
*IL-1β*	F: TGGGAAACAACAGTGGTCAGG
	R: CCATCAGAGGCAAGGAGGAA
*TNF-α*	F: GAGTGACAAGCCTGTAGCC
	R: CTCCTGGTATGAGATAGCAAA
*GAPDH*	F: CCGAGCTGAACGGGAAGCTCAC
	R: CCATGTAGGCCATGAGGTCCACC

GAPDH, glyceraldehyde 3-phosphate dehydrogenase; IL, interleukin; TNF, tumor necrosis factor; F, forward; R, reverse.

### 2.6 Immunoassay via enzyme-linked immunosorbent assay

The samples were obtained and analyzed as described by Prakash et al. (2024). The concentrations of cytokines, including IFN-β (424001, Thermo Fisher Scientific), interleukin (IL)-1β (SMLB00C, R&D Systems), IL-6 (SM6000B, R&D Systems), and tumor necrosis factor (TNF)-α (SMTA00B, R&D Systems), were quantified using ELISA kits according to the manufacturer's instructions. Briefly, serum samples were collected from the mice at designated time points and diluted as necessary. Each sample was added to the pre-coated wells, followed by the addition of detection antibodies and subsequent color development. The absorbance was measured using a microplate reader, and cytokine concentrations were calculated based on a standard curve (Prakash et al., 2024).

### 2.7 Histological analysis

Tissue samples, including lung, liver, heart, spleen, and kidney, were collected for histological analysis. The samples were rinsed with sterile saline and fixed in 4% paraformaldehyde for 24 h. These specimens were then processed following the hematoxylin and eosin (H&E) staining protocol (Su et al., 2024b). After undergoing dehydration, the tissues were sectioned into 4-μm slices and mounted on slides. These slides were then heated to 60°C for deparaffinization, rehydrated, and stained with H&E (Sigma-Aldrich, Burlington, MA, USA). Subsequently, excess dye was removed using ethanol and xylene, and coverslips were applied. For tissue, a semi-quantitative scoring system was used to assess inflammation, edema, hemorrhage, and alveolar septal thickening (Palumbo et al., 2017). Each parameter was scored on a 0- to 4-point scale, based on the severity of the injury observed in H&E-stained tissue sections. For inflammation, inflammatory cells were counted per field, and the other parameters were categorized based on the percentage of alveoli involved or septal thickness. The scores for each parameter were averaged to provide an overall injury score for each group.

### 2.8 Cytometric analysis

Flow cytometry was conducted in line with the established guidelines (Su et al., 2024a), utilizing the antibodies specified in Table 1. This included the process of erythrocyte lysis, followed by the resuspension of cells in 2% HyClone PBS solution for counting. Cell surface markers were labeled through a 30-min incubation with targeted antibodies at 4°C. Peripheral blood mononuclear cells were identified using Fixable Viability Stain 780 (FVS780) and the viability of cells was assessed. The analysis was executed using a FACSymphony™ A5 cytometer (BD Biosciences, CA, USA) and the data were analyzed using FlowJo software (v10.5.3; Ashland, OR, USA).

### 2.9 Collection of ascitic fluid samples

To evaluate the impact of AE and TIE on the peritoneal microbiome in septic mice, animals were anesthetized and then euthanized by cervical dislocation. The procedural area was disinfected using 75% alcohol. In a sterile setting, 1 mL of saline was infused into the peritoneum, followed by gentle peritoneal massage to enhance fluid recovery. Ascitic fluid was collected in Eppendorf centrifuge tubes for further analysis.

### 2.10 Peritoneal bacterial count

Serial dilution was performed on the ascitics fluid samples, with 100 μL from each dilution plated on agar and incubated at 37°C for 24 h. Finally, colony counting was performed based on the following formula:


$$\begin{array}{l} CFU/mL=\frac{colony\;number\;*\;dilution\;factor}{Inoculation\;volume\;\left(mL\right)}\; \end{array}$$

The number of colonies counted served as an indication of bacterial load.

### 2.11 Western blotting

Western blotting procedures were conducted according to established methods (Su et al., 2024c), which included steps such as protein extraction, electrophoresis, transfer to membranes, incubation with primary and secondary antibodies, washing, and chemiluminescent detection. Protein bands were quantitatively analyzed and their expression levels were presented as fold changes relative to control samples. The specific antibodies utilized are detailed in Table 1.

### 2.12 16S rRNA sequencing and bioinformatics analysis

16S rRNA in ascitic fluid samples was sequenced and analyzed at Beijing BioMarker Bioinformatics Technology Co., Ltd. (Beijing, China). DNA was extracted, and 16S rRNA sequencing was conducted according to established procedures outlined in a previous study (Su et al., 2024e). The Magnetic Soil and Stool DNA Kit (Cat. 4992738, Tiangen Biotech Co., Ltd.) was utilized for DNA extraction following the manufacturer's instructions. The complete 16S rRNA gene was amplified using the general primers 27F (5′-AGRGTTTGATYNTGGCTCAG-3′) and 1492R (5′-TASGGHTACCTTGTTASGACTT-3′), with the addition of PacBio barcode sequences unique to each sample. Library construction, sequencing, and data analysis were carried out using the PacBio Sequel II platform (Beijing BioMarker Bioinformatics Technology Co., Ltd.). The bioinformatics analysis was conducted using BMK Cloud (https://www.biocloud.net) following a published protocol. PICRUSt (Phylogenetic Investigation of Communities by Reconstruction of Unobserved States) was employed to predict the functional traits of the microbiome by using a phylogenetic approach (Su et al., 2024c).

### 2.13 Whole-genome sequencing analysis

Beijing BioMarker Bioinformatics Technology Co., Ltd. conducted RNA sequencing (RNA-seq) on the Illumina NovaSeq 6000 platform (Illumina, Inc., San Diego, CA, USA). The procedure followed an established protocol for comprehensive genomic sequencing analysis (Zhang et al., 2021).

### 2.14 Statistical analysis

Photoshop and Illustrator 2020 (Adobe, San Jose, CA, USA) along with ImageJ v1.8.0 (NIH, MD, USA) were used for preparing images. According to the guidelines provided on the GraphPad website (https://www.graphpad.com/guides/prism/latest/user-guide/citing_graphpad_prism.htm), GraphPad Prism (v8.0) was used to conduct statistical analyses using *t*-tests, one- and two-way ANOVA, and the Mantel–Cox (log-rank) test. Data are reported as mean ± SD, with statistical significance defined at *P* < 0.05.

## 3 Results

### 3.1 Therapeutic effect of AE and TIE treatment in mice with CLP-induced sepsis

**Figure 1:**
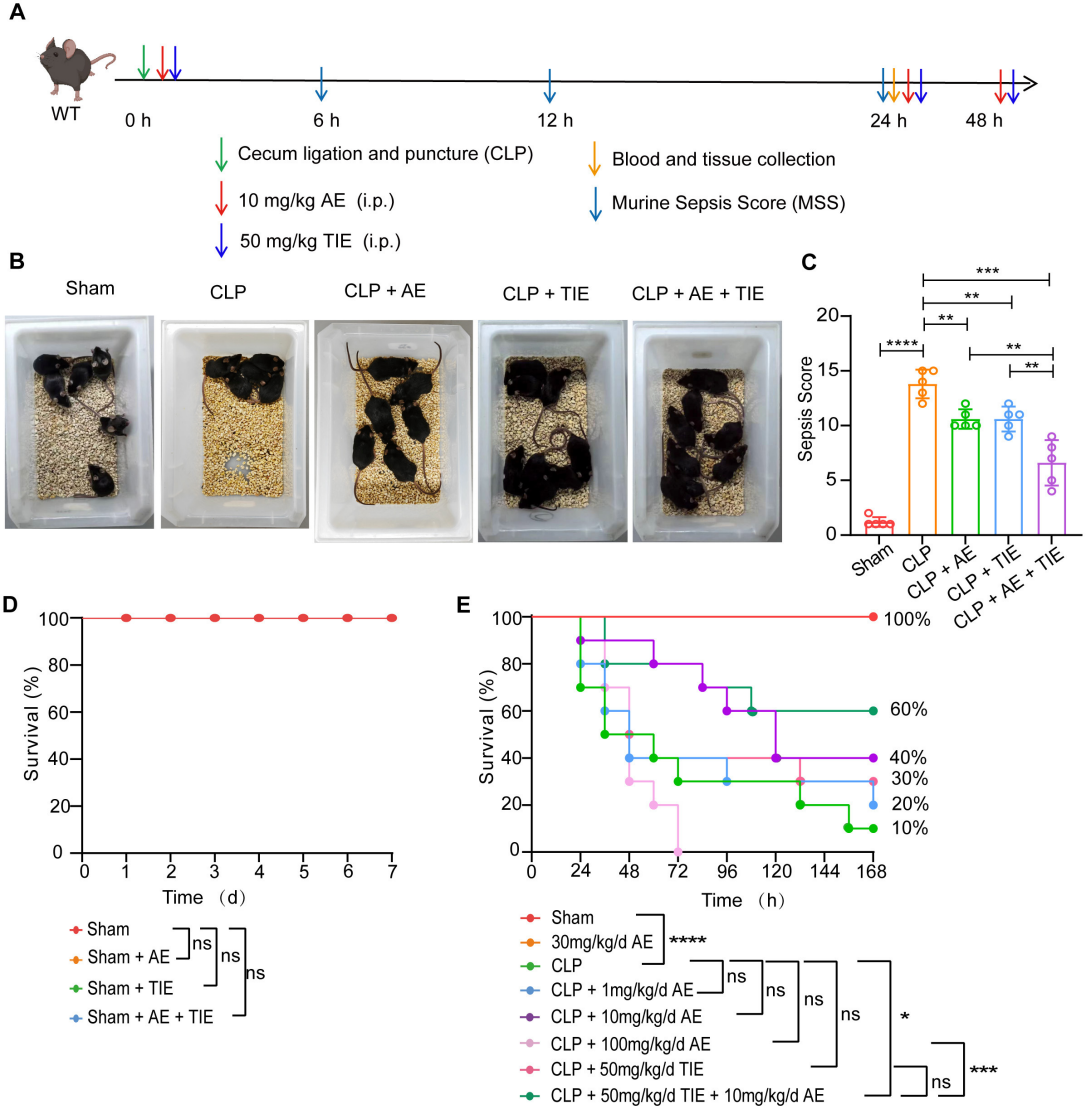
Combined therapeutic efficacy of aloe-emodin (AE) and TIENAM (imipenem and cilastatin sodium for injection, TIE) in a mouse model of CLP-induced sepsis. **(A)** Schematic representation of the experimental protocol. **(B)** Illustration of typical behavioral alterations observed in mice following a 12-h regimen of AE (10 mg/kg/day) and TIE (50 mg/kg/day) administration. **(C)** Assessment of the murine sepsis score (MSS) in septic mice following 12 h of concurrent AE and TIE treatment (*n* = 10). Data are expressed as mean ± SEM. Statistical analysis was conducted using ANOVA followed by Tukey's *post hoc* test. **P < 0.01, ***P < 0.001, and ****P < 0.0001. **(D)** Impact of AE and TIE administration on the survival of the sham group (*n* = 10). **(E)** Survival outcomes of septic mice subsequent to the treatment (*n* = 20). Survival analysis was performed using the Mantel–Cox test; *P < 0.05 and ***P < 0.001; ns, not significant. Sample sizes are indicated within parentheses.

The therapeutic potential of the combination of AE (10 mg/kg/day) and TIE (50 mg/kg/day) was evaluated using a CLP-induced sepsis mouse model (Figure 1A). Control groups received an equivalent volume of vehicle (corn oil) to match the treatment groups. The dosages were selected based on prior studies that demonstrated significant therapeutic effects at these levels. The rationale behind the choice of these specific concentrations and the administration route has been detailed in our previous publications (Su et al., 2023a, 2024d). Compared to the control group solely treated with CLP, the administration of AE, TIE, or both in significantly improved the health of the mice. The AE + TIE group exhibited notably enhanced outcomes, including active feeding, improved fur sheen, and no fur clumping, illustrating the most substantial benefits (Figure 1B). Post-treatment, there was a significant decrease in the MSS, with the AE + TIE group showing a remarkable reduction compared to the CLP control group (*P* < 0.0001; Figure 1C), underscoring the enhanced therapeutic impact of the combination therapy. In contrast, neither AE nor TIE alone significantly altered these health indicators in the sham-operated group (*P* > 0.05; Figure 1D). Furthermore, the survival rate in the AE + TIE group was significantly higher than in the CLP control group, with a survival rate of 75% vs. 10% (*P* < 0.0001; Figure 1E), highlighting the critical effectiveness of AE and TIE in managing CLP-induced sepsis in mice.

### 3.2 Reduction in inflammatory cytokine levels through AE and TIE treatment in CLP-induced sepsis mice

**Figure 2:**
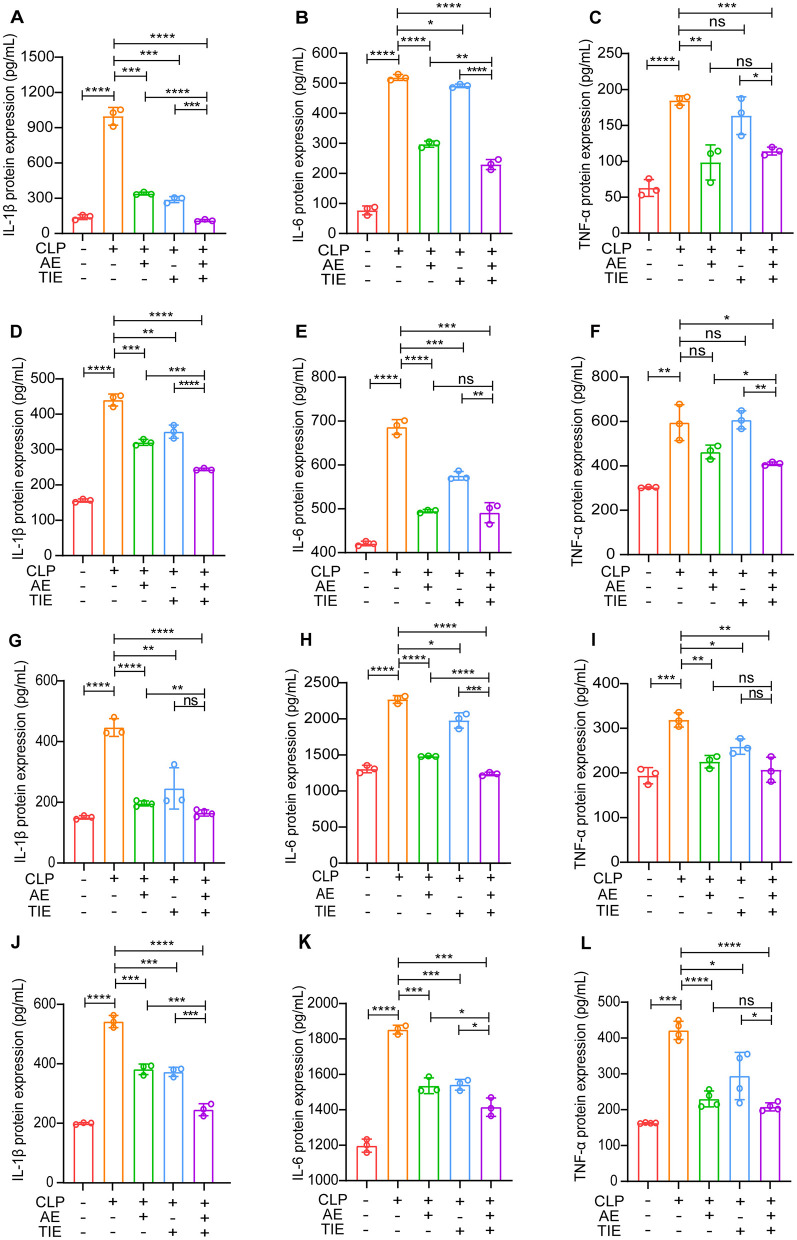
Modulation of inflammatory cytokines by AE + TIE in a CLP-induced sepsis mouse model. Levels of IL-1β, IL-6, and TNF-α were measured in **(A–C)** serum, **(D–F)** lung, **(G–I)** liver, and **(J–L)** kidney tissues after a 12-h treatment period, utilizing ELISA. Data are represented as mean ± SEM. Statistical analysis was performed using ANOVA followed by Tukey's *post hoc* tests (*n* = 3). *P < 0.05, **P < 0.01, ***P < 0.001, and ****P < 0.0001; ns, not significant.

The regulatory effect of AE and TIE on inflammatory cytokine production was assessed using a mouse model of CLP-induced sepsis. This investigation concentrated on how AE and TIE influence cytokine concentrations in various organs such as the serum, lungs, liver, and kidneys. The administration of AE and TIE did not yield statistically significant effects on these parameters in the sham group (*P* > 0.05, Supplementary Figure 1). However, the combined treatment notably decreased the heightened serum concentrations of IL-1β, IL-6, and TNF-β, demonstrating significant reductions (*P* < 0.01, Figures 2A–C). Cytokine levels also consistently declined in the tissues of the lungs, liver, and kidneys, validating the broad and effective nature of the treatment (Figures 2D–L).

**Figure 3:**
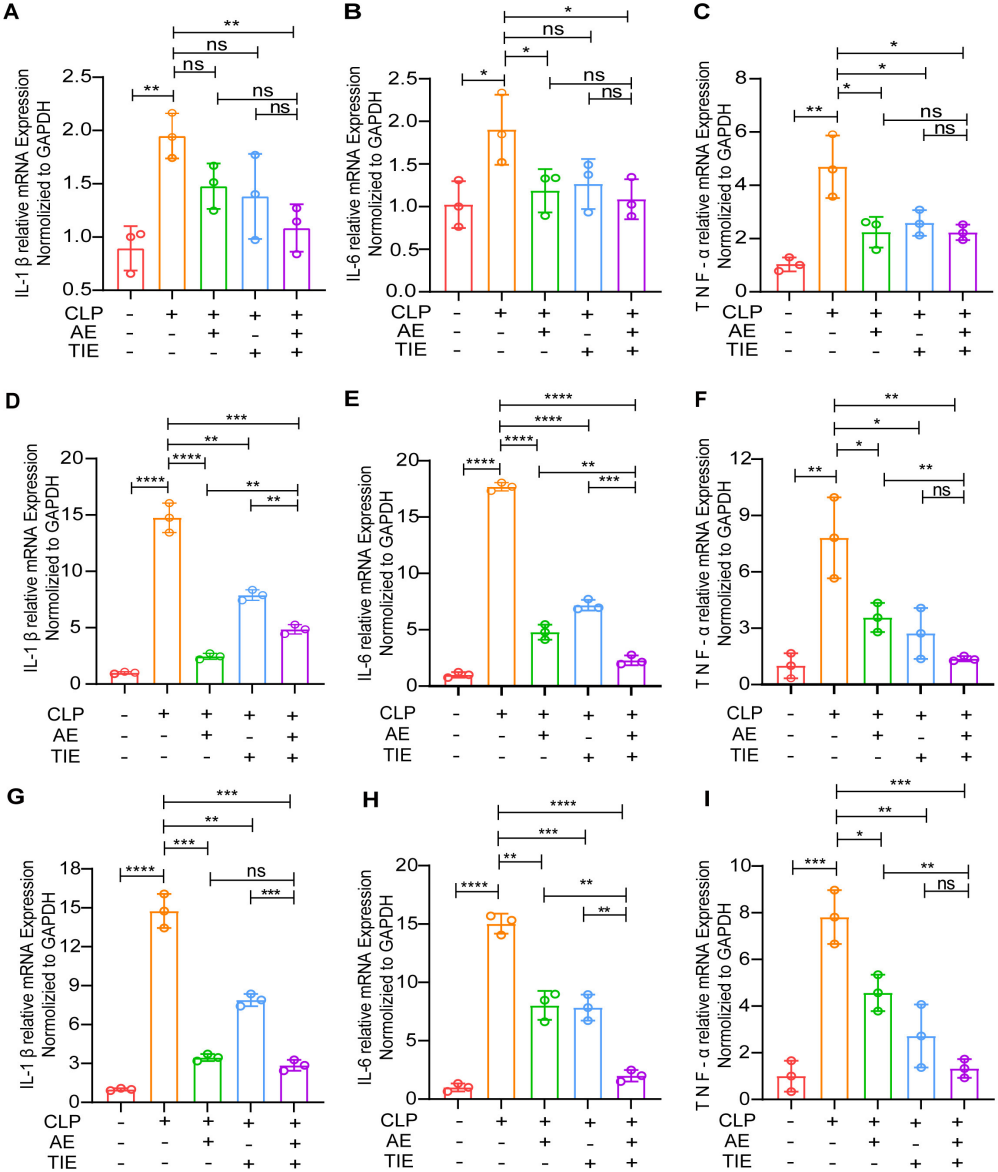
Analysis of pro-inflammatory cytokine expression in mouse model of CLP-induced sepsis following AE and TIE administration. This Figure quantifies the expression levels of IL-6, IL-1β, and TNF-α in lung **(A–C)**, liver **(D–F)**, and kidney **(G–I)** tissues of mice subjected to sepsis via CLP and treated with AE (10 mg/kg/day) and TIE (50 mg/kg/day) for 12 h. Quantitative RT-PCR was employed for measurement. Data are presented as mean ± SEM. Statistical significance was determined using ANOVA and Tukey's *post hoc* tests (*n* = 3). *P < 0.05, **P < 0.01, ***P < 0.001, and ****P < 0.0001; ns, not significant.

Subsequent RT-qPCR analysis after CLP induction showed a marked decrease in IL-6, IL-1β, and TNF-α expression in the lung, liver, and kidney tissues of the AE + TIE group, markedly exceeding the results seen in the CLP-only group (*P* < 0.05, Figure 3). These results strongly highlight the potent anti-inflammatory effects of the AE and TIE combination, emphasizing its potential as an effective therapeutic approach for combating CLP-induced sepsis in mice.

### 3.3 AE and TIE treatment improved CLP-induced pathology

**Figure 4:**
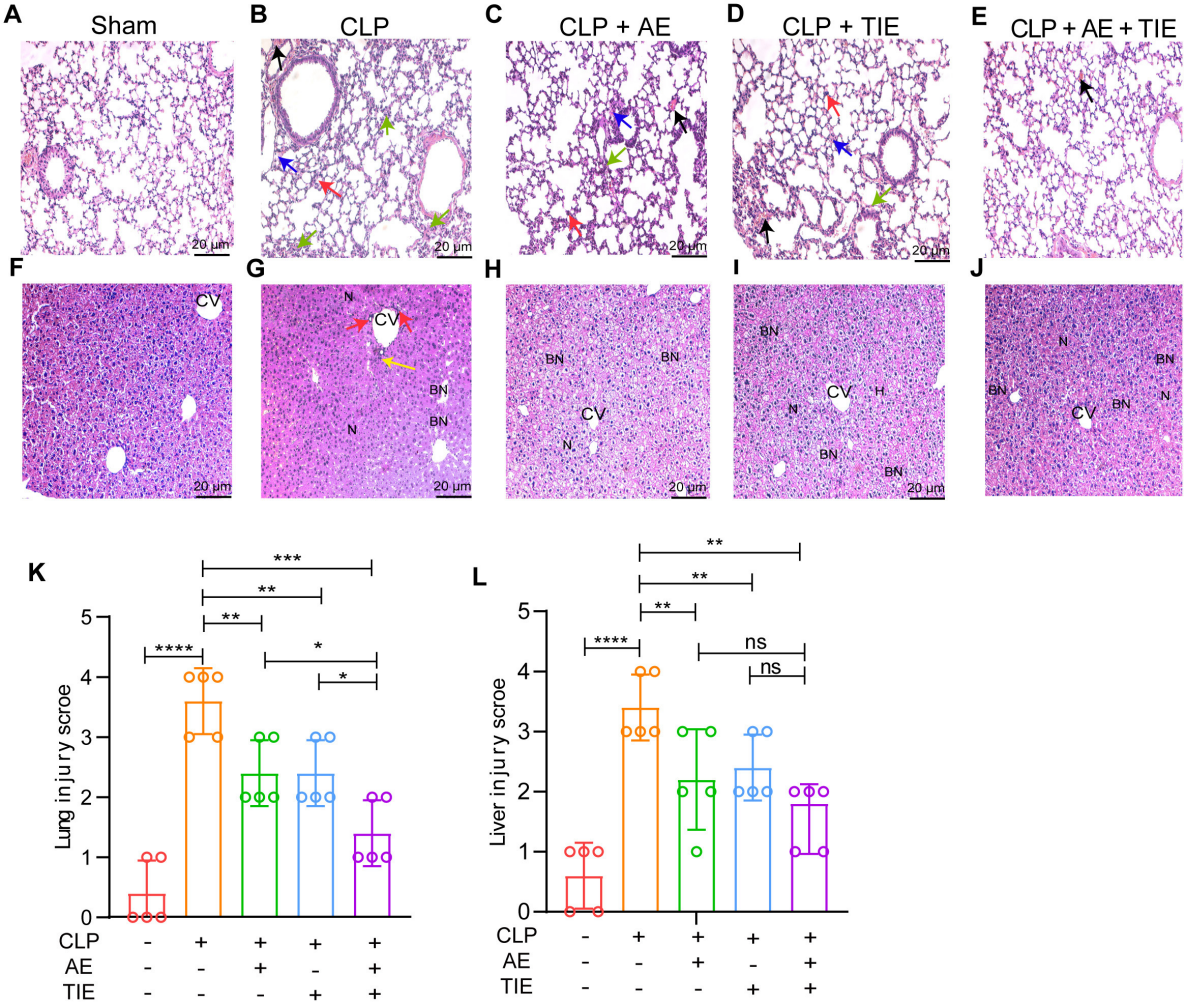
AE + TIE effects on lung and liver in a CLP-induced sepsis mouse model. Microscopic images of lung **(A–E)** and liver **(F–J)** lesions stained with hematoxylin and eosin (H&E), observed at 200× magnification. Indicators of tissue damage include pulmonary edema (red arrows), infiltration by inflammatory cells (blue arrows), thickening of alveolar walls (black arrows), and cardiomyocyte disruption (yellow arrows). Pathological scoring for lung **(K)** and liver **(L)** tissues. Data are depicted as mean ± SEM. Statistical analysis was conducted using Tukey's *post hoc* test following one-way ANOVA (*n* = 5). *P < 0.05, **P < 0.01, ***P < 0.001, and ****P < 0.0001; ns, not significant. Scale bars: 50 μm.

Following the administration of the treatment, tissue samples were analyzed using H&E staining at 12 h post-treatment to assess injury severity. The combination therapy of AE and TIE notably diminished lung injury, as evidenced by the reduced alveolar wall thickening and fewer occurrences of hemorrhages (Figures 4A–E). In the liver, the AE + TIE group displayed significant injury reduction, characterized by diminished inflammatory cell infiltration, decreased swelling, and better hepatocyte organization, compared to the CLP control group (Figures 4F–J). Pathological evaluations further confirmed the significant effectiveness of AE and TIE combination treatment in reducing tissue damage across critical organ systems (*P* < 0.01; Figures 4K, L). Altogether, these findings underscore the considerable potential of AE and TIE combination therapy in alleviating the adverse effects of CLP-induced sepsis on respiratory, hepatic, and cardiovascular functions.

### 3.4 AE and TIE treatment altered immune cell profiles in mice with CLP-induced sepsis

**Figure 5:**
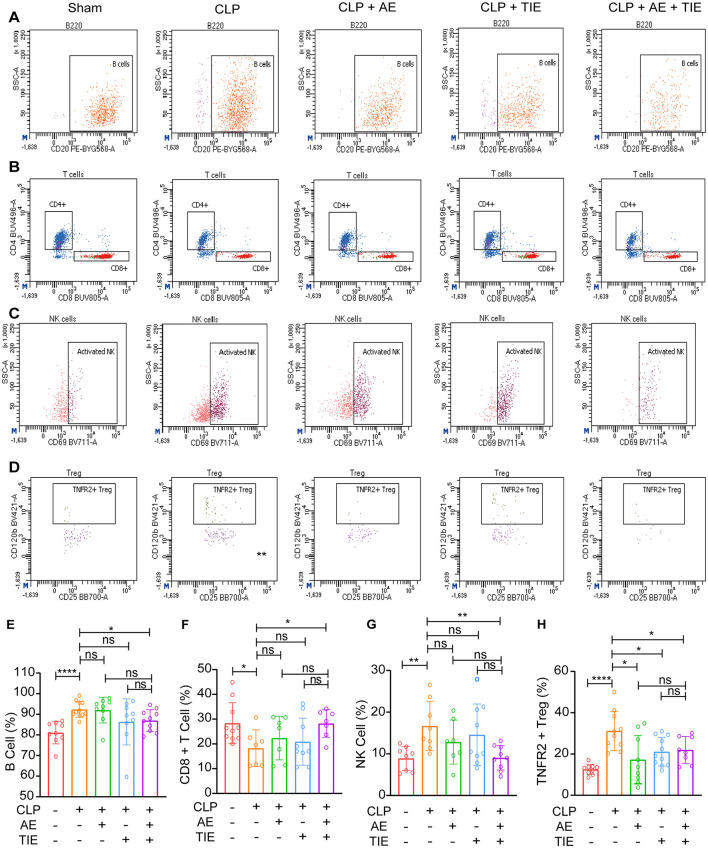
Impact of AE and TIE treatment on peripheral blood immune cells in septic mice. **(A)** Gating plots showing B cells. **(B)** Gating plots depicting CD8^+^ T cells. **(C)** Gating plots illustrating NK cells. **(D)** Gating plots demonstrating TNFR2^+^ T_reg_ cells. Quantitative analysis of **(E)** B cells, **(F)** CD8^+^ T cells, **(G)** NK cells, and **(H)** TNFR2^+^ T_reg_ cells. Data are expressed as mean ± SEM. Statistical analysis was performed using Tukey's *post hoc* test following one-way ANOVA (*n* = 5-10). *P < 0.05, **P < 0.01, and ****P < 0.0001; ns, not significant.

Using flow cytometry to evaluate immune responses 12 h post-treatment, we observed a notable decrease in B cell activation in the AE + TIE group compared to the TIE-only cohort (*P* < 0.05, Figures 5A, E). Additionally, CD8^+^ T cell activation was significantly increased after treatment with combined AE + TIE (*P* < 0.05, Figures 5B, F). Although treatments with AE and TIE alone did not significantly alter the NK cell count relative to that in the CLP model, AE + TIE treatment significantly decreased it (*P* < 0.05, Figures 5C, G). Notably, TNFR2^+^ T_reg_ cell counts were considerably elevated in the combination therapy group, suggesting an important immunomodulatory role (*P* < 0.05, Figures 5D, H). Despite these notable changes, no significant differences were observed in the CD4^+^ T cell and activated NK cell counts under septic conditions (*P* > 0.05, Supplementary Figure 2). These results highlight the selective effects of AE and TIE treatments on specific immune cell activations, demonstrating their targeted modulation of immune responses in sepsis-induced mice.

### 3.5 AE and TIE treatment significantly reduces the bacterial load in the peritoneal cavity of mice with CLP-induced sepsis

**Figure 6:**
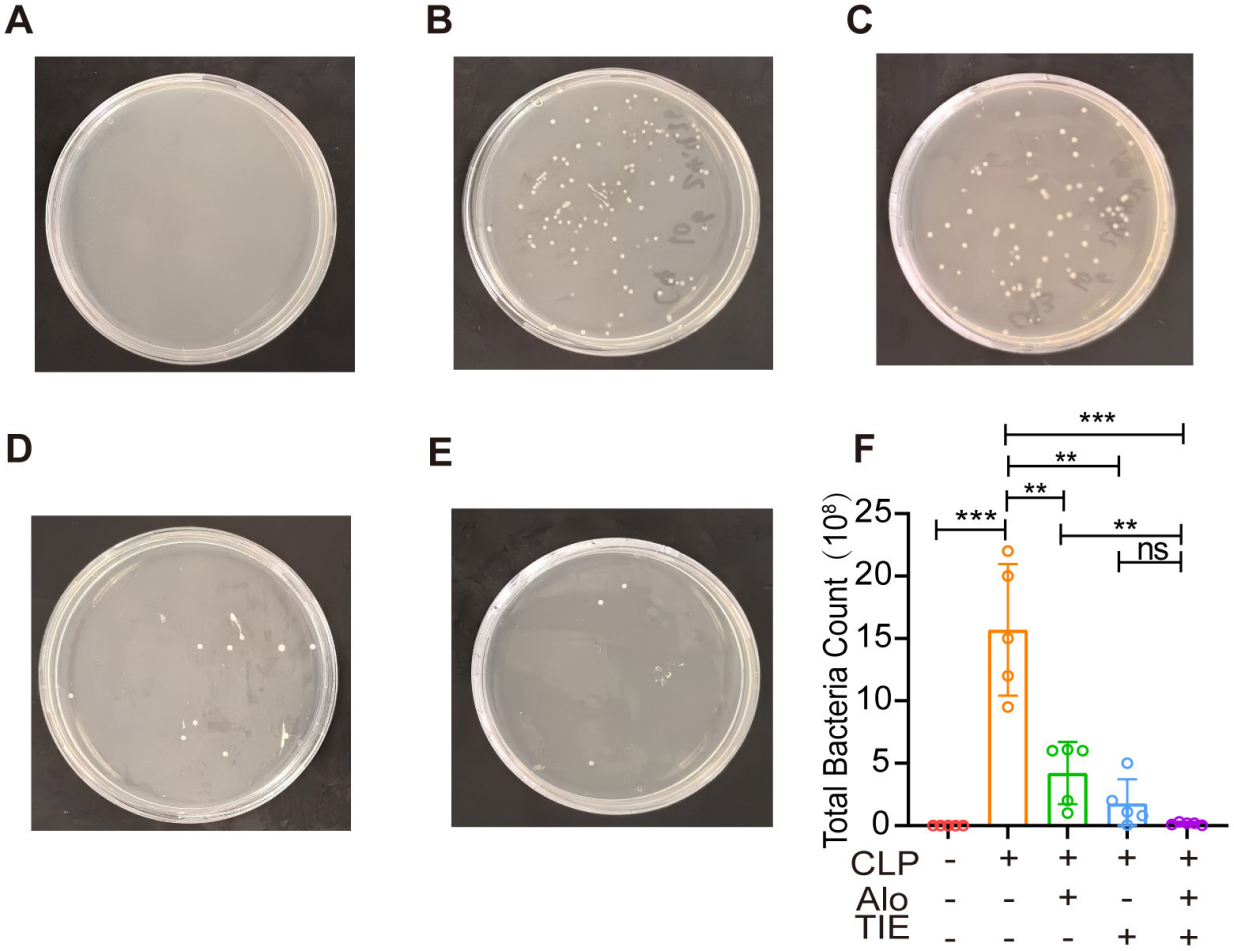
Effects of AE and TIE treatment on intraperitoneal bacterial contents of mouse model of sepsis. **(A–E)** Bacterial cultures obtained from ascitic fluids using the spreading method. **(A)** Sham group, **(B)** CLP group, **(C)** AE group, **(D)** TIE group, **(E)** AE + TIE group. **(F)** Quantification of bacterial load analyzed using Tukey's *post hoc* test following one-way ANOVA. Data are presented as mean ± SEM (*n* = 5). *P < 0.05, **P < 0.01, ***P < 0.001, and ****P < 0.0001; ns, not significant.

Our evaluation focused on the capability of AE and TIE to diminish bacterial colonies. According to Figure 6, although all groups showed some level of bacterial presence, the CLP group presented a notably higher colony count (*P* < 0.0001). In contrast, treatments with AE, TIE, and especially the combined AE + TIE therapy exhibited substantial reductions in bacterial growth, with TIE alone demonstrating the most significant decrease (*P* < 0.01).

### 3.6 AE and TIE treatment modulates microbial equilibrium in the peritoneal cavity of mice with CLP-induced sepsis

**Figure 7:**
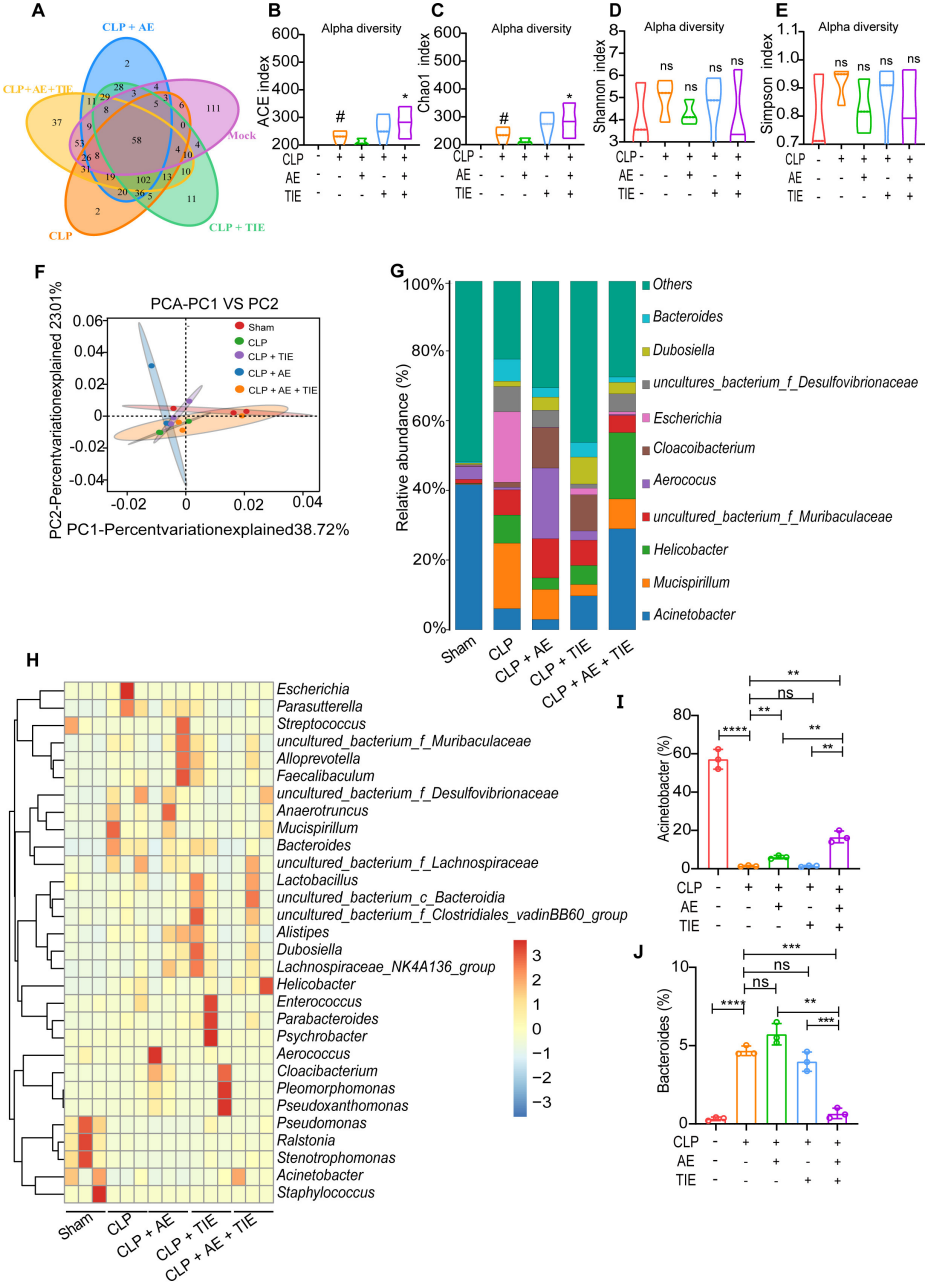
Restoration of peritoneal microbiota balance in a sepsis mouse model by AE + TIE. **(A)** A Venn diagram illustrates the overlap of operational taxonomic units (OTUs) of intestinal microbes among treatment groups, revealing interplay between treatments. **(B–E)** Presentation of alpha diversity metrics, including the **(B)** Ace index, **(C)** Chao1 index, **(D)** Shannon index, and **(E)** Simpson index. **(F)** Beta-diversity assessed through UniFrac Adonis Principal Components Analysis (PCA) plots, highlighting variance in the microbial community. **(G)** Overview of genus-level peritoneal flora composition. **(H)** Heat maps visually depict genus distinctions among groups, featuring key genera such as **(I)** *Acinetobacter* and **(J)** *Bacteroides*. Heat maps utilize row-normalized Z-values for clarity. Statistical analyses, conducted via ANOVA and Tukey's *post hoc* test, reveal significant differences (*n* = 5). **P < 0.01, ***P < 0.001, and ****P < 0.0001; ns, not significant. # means *P* < 0.05, against the sham group, *P < 0.05, against the CLP group.

Employing advanced 16S RNA sequencing, our study assessed changes in the microbial landscape post-treatment. Analysis using a Venn diagram and subsequent alpha- and beta-diversity assessments indicated unique microbial community structures among the groups (Figure 7A). To investigate the impact of AE + TIE on intestinal flora diversity and abundance in septic mice, we analyzed the alpha diversity at the operational taxonomic unit (OTU) level. This included the Ace, Chao1, Shannon, and Simpson indices, where Ace and Chao1 reflect species richness, and Shannon and Simpson indicate species diversity. The results showed that both the Ace and Chao1 indices significantly increased in the CLP group compared with those in the blank group (*P* < 0.01), with similar trends for the Shannon and Simpson indices (*P* < 0.01). In the CLP + AE + TIE group, the Ace and Chao1 indices further increased compared with those in the CLP group (*P* < 0.01) (Figures 7B–F). These findings suggest that CLP enhances intestinal flora diversity and abundance, and the combination of 10 mg/kg AE with 50 mg/kg TIE effectively boosts these metrics in CLP septic mice. At the genus level, *Acinetobacter, Mucispirillum, Helicobacter, Escherichia*, and *Bacteroides* were predominant. *Acinetobacter* levels were lower in the CLP model group compared with those in the Sham group, while *Bacteroides* levels were higher. However, compared with the CLP model groups, *Acinetobacter* levels were higher and *Bacteroides* levels were lower in the CLP +AE +TIE groups. Therefore, the AE and TIE treatments significantly modified the levels of pathogenic and beneficial bacteria, enhancing microbial homeostasis in the peritoneal cavity (Figures 7G–J).

Furthermore, using linear discriminant analysis effect size and predictive functional profiling, we elucidated the broad-spectrum impacts of these treatments on microbial gene function (Supplementary Figure 3). Using reconstruction of unobserved states analysis, the impact of combined AE + TIE therapy was also assessed on gut microbiome function. These analyses highlighted pathways involved in environmental adaptation, immune diseases, and signal transduction (Supplementary Figure 4), offering insights into the complex mechanisms through which AE and TIE therapies recalibrate microbial dynamics and community functions, thereby enhancing their therapeutic efficacy against sepsis-induced dysbiosis.

This comprehensive evaluation confirms the multifaceted role of AE and TIE therapy in restoring microbial balance, curtailing pathogenic bacterial overgrowth, and fostering beneficial bacterial proliferation. This positions the combined therapy as a potent therapeutic strategy against sepsis-induced microbial dysregulation.

### 3.7 AE and TIE treatment suppresses CLP-induced inflammatory response and apoptosis

**Figure 8:**
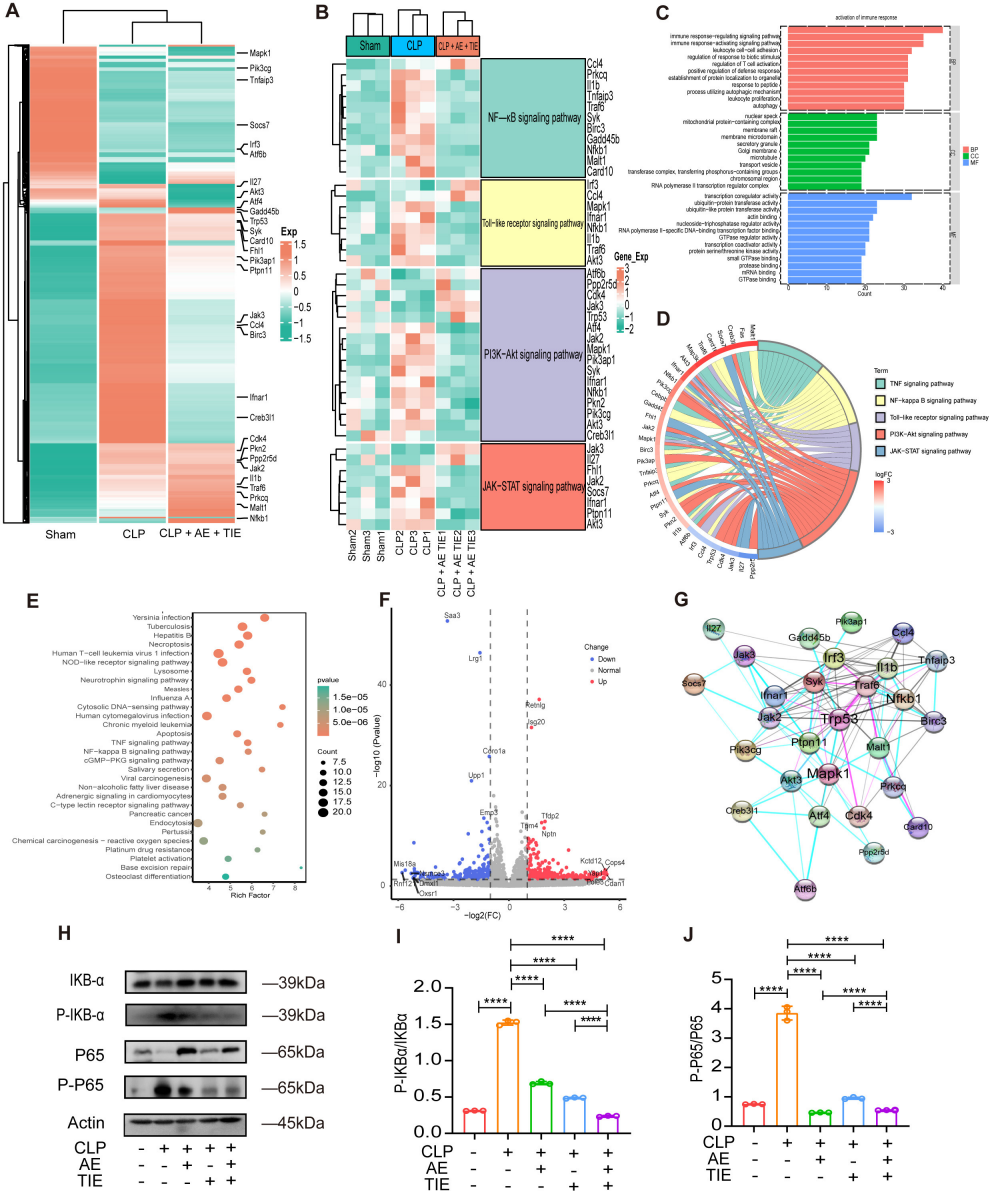
Impact of AE + TIE on NF-κB pathways in CLP-induced septic mice. **(A–G)** Gene profile analysis in septic mice following CLP with AE + TIE treatment. **(A)** Clustered heatmap illustrating gene expression in Sham, CLP, and AE + TIE-treated septic mice. **(B)** Heatmap of differentially expressed genes (DEGs) in Sham, CLP, and AE + TIE-treated septic mice. **(C)** GO enrichment analysis of DEGs (*P* < 0.05). **(D)** The GO chord plot revealed the top 6 significantly enriched GO pathways. **(E)** KEGG analysis of DEGs in CLP and AE + TIE-treated septic mice (CLP vs. AE + TIE treatment). **(F)** Volcano plot displaying DEGs following AE + TIE treatment compared to CLP-induced septic mice (CLP vs. AE + TIE treatment; downregulated genes, blue; upregulated genes, red). **(G)** Identification of protein interaction networks among TNF, NF-κB, and PI3K-Akt using the STRING database. **(H)** Western blotting analysis of IKBα, p- IKBα, NF-κB (p65), and p-NF-κB (p-p65) protein levels in lung homogenates after AE + TIE treatment. **(I, J)** Western blotting assessment of protein expression levels of IKBα, p- IKBα, NF-κB (p65), and p-NF-κB (p-p65) in lung homogenates after AE + TIE treatment. Densitometric analysis of bands was performed using ImageJ software. Data are presented as mean ± standard deviation. ANOVA and Tukey's *post hoc* test were performed to analyze the data (*n* = 3). ****P < 0.0001.

Comprehensive RNA-seq of the entire genome elucidated the molecular pathways responsible for the protective effects of combined AE + TIE treatments in sepsis. Heatmaps in Figures 8A, B illustrate changes in gene expression, allowing the identification of differentially expressed genes. The combination therapy treatments attenuated the expression of key genes across multiple pathways, including the NF-κB (IL-1β and Nfkb1), Toll-like receptor (Mapk1 and IFNAR1), PI3K-Akt (Aft4 and Syk), and JAK-STAT (Fhl1 and Jak2) pathways (Figures 8A, B). Enrichment analyses of differentially expressed genes through Gene Ontology and Kyoto Encyclopedia of Genes and Genomes highlighted the significant impact of AE + TIE treatment. CLP significantly activated the TNF, NF-κB, Toll-like receptor, PI3K-Akt, JAK-STAT, and TNF signaling pathways. In contrast, AE + TIE treatment effectively reduced inflammatory responses (Figures 8C–E). The volcano plot and Venn diagram revealed the induction of Retnig, Isg20, Tfdp2, Nptn, and Tpm4 by CLP and the suppression of Saa2, Lrf1, Coro1a, Upp1, and Emp3 by AE+TIE treatment (Figure 8F). STRING analysis displayed the functional interaction between TRP53, NF-κB, MAPK, JAK2, IFNAR1, IRF3, TNF, and CDK4 proteins (Figure 8G).

Moreover, western blot analysis of lung tissue was performed to evaluate the impact of AE + TIE therapy on sepsis induced by CLP, indicated notable inflammation. The combination treatment significantly decreased NF-κB activation, as evidenced by the reduced NF-κB (p65) and IKBα phosphorylation relative to that under CLP conditions (*P* < 0.001, Figures 8H–J). Overall, the combination treatment mitigates inflammatory responses triggered by CLP, thus having potential as a novel therapeutic approach for managing sepsis.

## 4 Discussion

This study is the first to investigate the effects of combined AE and TIE treatment on mice with CLP-induced sepsis. Our findings indicate a significant improvement in survival rates, with septic mice treated with AE + TIE (10 + 50 mg/kg/day) showing a 60% survival rate at 168 h compared to only 10% in the CLP model group. The combined treatment effectively suppressed the expression of inflammatory cytokines, such as, IL-6, IL-1β, and TNF-α, in vital organs, including the lungs, liver, and kidneys, at both the transcription and translation levels. Additionally, AE treatment significantly improved tissue damage, reduced inflammatory cell infiltration, and mitigated organ injury caused by sepsis. The AE + TIE regimen significantly decreased B, NK, and TNFR2^+^ T_reg_ cell counts, while substantially increasing that of CD8^+^ T cells. Furthermore, this combination therapy effectively reduced the bacterial load within the peritoneal cavity and altered the composition of specific bacterial groups post-inflammation. Moreover, the AE + TIE therapy significantly decreased the abundance of harmful bacteria, such as *Bacteroides*, highlighting the complex interplay of AE + TIE therapy and sepsis-induced inflammatory responses.

In our previous studies, we conducted a comprehensive analysis of the toxicity profile of AE. Our findings, as detailed in earlier publications (Su et al., 2023a), demonstrate that AE does not cause significant tissue damage when administered alone, as confirmed by histological examination (HE). Moreover, in a study involving mice with CLP-induced sepsis, AE administration did not lead to notable changes in hematological parameters compared to the control group, indicating that AE does not adversely impact blood parameters under septic conditions. *In vitro* experiments using murine RAW264.7 cells further revealed that even at high doses, AE did not compromise cellular viability. Together, these results highlight the safety and non-toxic nature of AE in the evaluated contexts. Additionally, TIENAM was administered in this study at a dose of 50 mg/kg/day, which is lower than the clinically recommended dose and does not exhibit significant toxicity, consistent with established clinical data. Furthermore, our group has previously explored the combination of Aloin with TIENAM (Su et al., 2024f) and ulinastatin with TIENAM (Su et al., 2024d) in the treatment of CLP-induced sepsis, further supporting the safety profile of these therapeutic regimens.

In sepsis, monocytes and neutrophils are crucial in initiating a “cytokine storm” by releasing inflammatory cytokines and promoting lymphocyte proliferation, which can exert cytotoxic effects (Weber et al., 2015; Bénard et al., 2021). Treatments targeting apoptotic cells have been shown to significantly mitigate this cytokine storm (Karbian et al., 2020). Therefore, effectively controlling sepsis requires managing inflammation and cell death levels (Su et al., 2023c). While AE and TIE individually reduce the levels of IL-6, IL-1β, and TNF-α in septic mice, their combined action significantly enhances these effects. This synergistic strategy not only reduces cytokine production but also adjusts the immune response. It counteracts sepsis-induced apoptosis in CD4^+^ T cells, which weakens defense mechanisms against pathogens, exacerbates bacterial dissemination, and intensifies inflammation, thereby accelerating sepsis progression (Gonnert et al., 2012). Additionally, it restricts the ability of CD8^+^ T cells to eradicate infected cells (Gouel-Chéron et al., 2012).

During sepsis, neutrophils interact with endothelial cells, migrate to inflamed sites, and participate in pathogen engulfment, simultaneously releasing antimicrobial agents (Shen et al., 2017). Encountering cytokines or pathogens, monocytes and macrophages phagocytose these invaders and initiate antigen presentation, leading effector T cells to prompt macrophages to release fibrosis-inducing agents (Hou et al., 2015). However, sepsis hampers the maturation and activation of dendritic cells, which impedes the rapid recruitment of innate immune cells such as monocytes, NK cells, and granulocytes. Additionally, sepsis-induced immunosuppression results in metabolic dysfunction in monocytes, marked by diminished activities like glycolysis and fatty acid oxidation (Cheng et al., 2016). While NK cells in sepsis generate substantial levels of INF-γ, they are compromised in supporting the Th1 immune responses essential for combating bacterial infections (Guo et al., 2018).

The AE + TIE combination distinctly alters the immune landscape in septic mice by reducing B, NK, and TNFR2^+^ T_reg_ cell numbers. Compared to TIE alone, the combined treatment significantly diminishes B and NK cell numbers, showcasing how AE modifies the effects of TIE on immune cell populations.

The combination therapy of AE and TIE has shown promise in fostering a favorable balance of intestinal microbiota while restraining the growth of pathogenic bacteria in septic mice. TIE, renowned for its wide-ranging antimicrobial efficacy, functions by stabilizing β-lactamase, rendering it a favored option for treating bacterial infections (Ding et al., 2019). Nonetheless, the increasing occurrence of carbapenem-resistant *Klebsiella pneumoniae* during septic episodes presents a formidable obstacle (Campanella and Gallagher, 2020). Additionally, the extended use of TIE can lead to bacterial resistance, and high TIE doses may impair stomach, liver, and kidney function, potentially causing muscle spasms and mental health issues (Heo, 2021). The chemical structure of AE is intricately linked to its pharmacological effects. The anthraquinone structure and the presence of a ketone functional group are responsible for its anti-inflammatory and antimicrobial properties (Weber et al., 2015; Karbian et al., 2020). AE has demonstrated significant efficacy in inhibiting pathogens such as *Acinetobacter baumannii, Candida albicans*, and *Staphylococcus aureus* (Arosio et al., 2000).

AE has been demonstrated to exert anti-inflammatory effects by inhibiting the activation of Toll-like receptor 2-mediated NF-κB and MAPK signaling pathways in LPS-induced macrophages. This inhibition is reflected by decreased mRNA levels of inducible nitric oxide synthase (iNOS), IL-6, and IL-1β (Wang P. et al., 2021; Wang Y. et al., 2021). Furthermore, AE reduces the secretion of high-mobility group box 1 protein by restoring the levels of endothelial-related proteins, such as zonula occludens-1 and−2, thus preventing the activation of the NLRP3 inflammasome, which is controlled by NLRP1 ubiquitination. This mechanism effectively reduces the inflammatory response (Dong et al., 2020). AE also inhibits cell death by reducing the levels of caspase-3, and improves outcomes in subarachnoid hemorrhage by affecting the NF-κB and cyclic adenosine monophosphate/protein kinase A/responsive element-binding protein pathways (Lin et al., 2022). Modulating these pathways prevents the production of inflammatory cytokines and nerve cell death caused by hemorrhage (Jiang et al., 2021). These findings support further investigation of AE as a potential treatment for preventing and managing sepsis. Notably, the antimicrobial activity of AE against *Staphylococcus epidermidis* and other Gram-positive pathogenic species (Li et al., 2021) suggests that the antibacterial effects of the AE + TIE combination potentially exceed those of the individual treatments.

PICRUSt analysis revealed that AE + TIE therapy significantly increased the expression of genes associated with nucleotide metabolism, replication, repair, energy metabolism, cellular proliferation, and death, suggesting a correlation between functional shifts in the microbiome and immune modulation in septic mice.

Correlation analyses unveiled distinct roles of microbes in inflammatory responses. TIE application alone augmented Helicobacter levels, which were subsequently diminished by the AE + TIE combination, underscoring the potential of AE in offsetting the microbial constraints of TIE. Additionally, *Bacteroides*, recognized for its pro-inflammatory cytokine-inducing properties (Nakamura et al., 2017), displayed reduced abundance in the AE + TIE-treated groups, highlighting the effectiveness of this therapy in microbial and inflammatory regulation.

While the concurrent administration of AE and TIE presents evident benefits in sepsis management, it also poses potential risks and challenges. Specifically, there is a possibility of drug interactions between AE and TIE affecting drug metabolism and elimination, thereby increasing susceptibility to adverse reactions. Hence, vigilant monitoring of drug concentrations and adverse effects is crucial during the utilization of this combination therapy. Further research is needed to clarify the specific roles of individual microbiota members and their metabolites in reducing sepsis-induced inflammation. The complex relationship between microbiota and sepsis requires additional investigation, possibly using germ-free models or microbiota transplants, to understand the underlying mechanisms.

## 5 Conclusions

This study underscores the synergistic advantages of combining AE with TIE, while addressing TIE-associated limitations and broadening the therapeutic applications of AE. By leveraging the distinct mechanisms of action of both AE and TIE, this innovative combination therapy offers a more comprehensive approach to combating sepsis than either treatment alone. Continued research into this novel treatment strategy has the potential to advance sepsis management and improve patient outcomes.

## Data Availability

The data presented in this study are deposited in the National Bioinformation Center (CNCB) repository, accession number CRA021008. For more information regarding our data policies, refer to our guidelines. The link to access the data is https://bigd.big.ac.cn/gsa/browse/CRA021008.
