# Supplementary material for: Aloe-emodin plus TIENAM ameliorate cecal ligation and puncture-induced sepsis in mice by attenuating inflammation and modulating microbiota
Source: Front Microbiol. 2024 Dec 12;15:1491169. doi: 10.3389/fmicb.2024.1491169 (PMC11669710; doi:10.3389/fmicb.2024.1491169)
Supplement: Supplementary file 1 [file Data_Sheet_1.docx]

**Aloe-emodin plus TIENAM ameliorate cecal ligation and puncture-induced sepsis in mice by attenuating inflammation and modulating microbiota**

Jingqian Su^1, †,^ *, Xiaohui Deng ^1, †^, Shan Hu^1^, Xinrui Lin^1^, Lian Xie^1^, Hui Ye^1^, Congfan Lin^1^, Fen Zhou^1^, Shun Wu^1^, Liling Zheng^2,^ *

^1^ Fujian Key Laboratory of Innate Immune Biology, Biomedical Research Center of South China, College of Life Science, Fujian Normal University, Fuzhou, Fujian Province 350117, PR China.

^2^ First Hospital of Quanzhou Affiliated to Fujian Medical University, Quanzhou, Fujian, China, 250 East Street, 362000, PR China.

*** Corresponding Author:**

Jingqian Su

Tel: +86-0591-22868201

E-mail: [sjq027@fjnu.edu.cn](mailto:sjq027@fjnu.edu.cn)

Liling Zheng

Tel: +86-18960203288

E-mail: zll@fjmu.edu.cn

^†^These authors contributed equally to this work.

**Supplementary Information:**

1. Supplementary Figures S1~S5

**
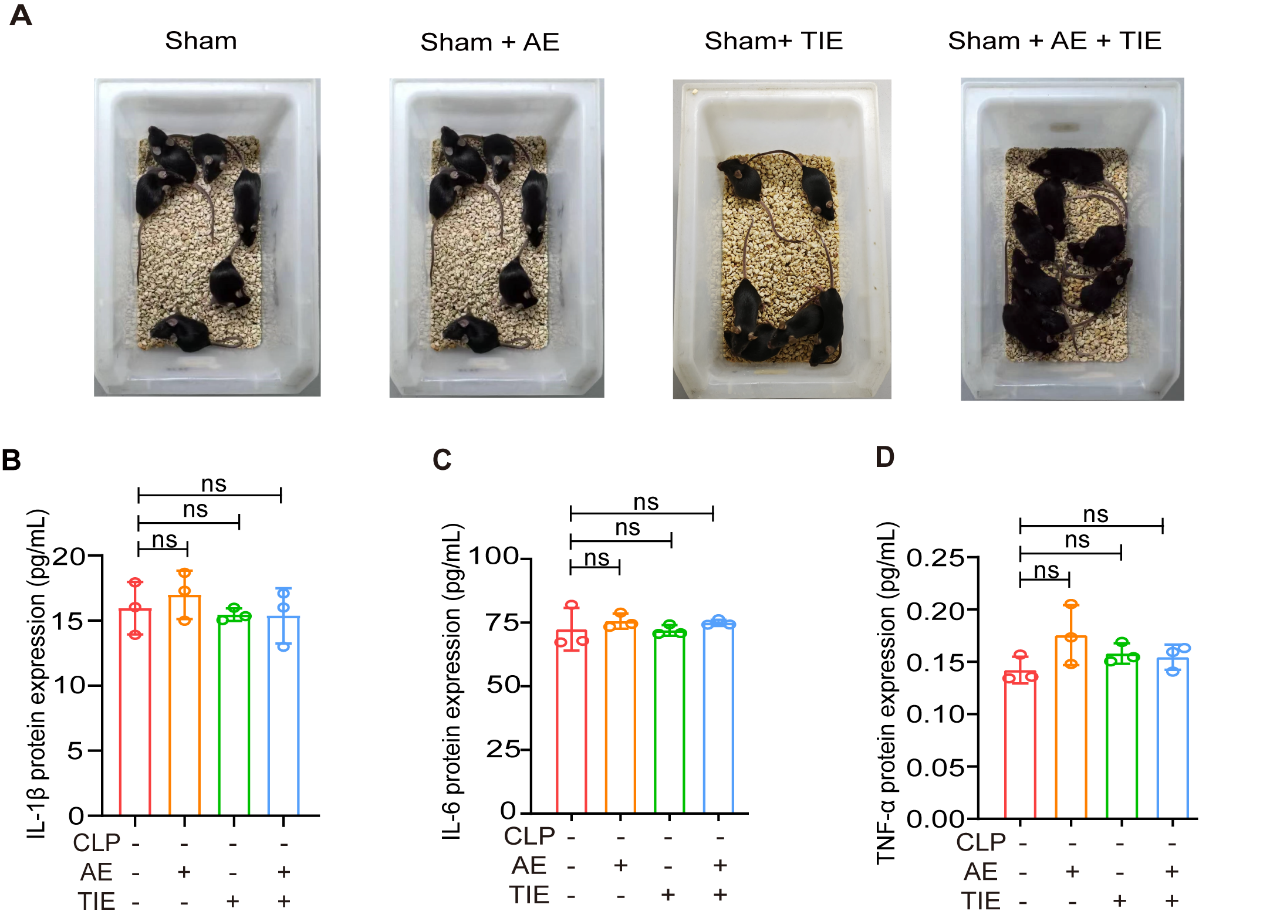
**

**Fig. S1. Effect of aloe-emodin (AE) and TIENAM (imipenem and cilastatin sodium for injection, TIE) administration on expression levels of inflammatory factors in the Sham group.** (A) Illustration of typical behavioral alterations. (B-D) Influence of AE and TIE administration on the serum expression levels of inflammatory factors IL-6 (B), IL-1β (C) and TNF-α (D)in the sham, sham + AE, sham + TIE, and sham + AE + TIE groups. Data are presented as mean ± SEM (*n* = 3). Statistical significance was determined using ANOVA and Tukey’s *post hoc* tests. ns, not significant (*P* > 0.05). vs. the sham group.


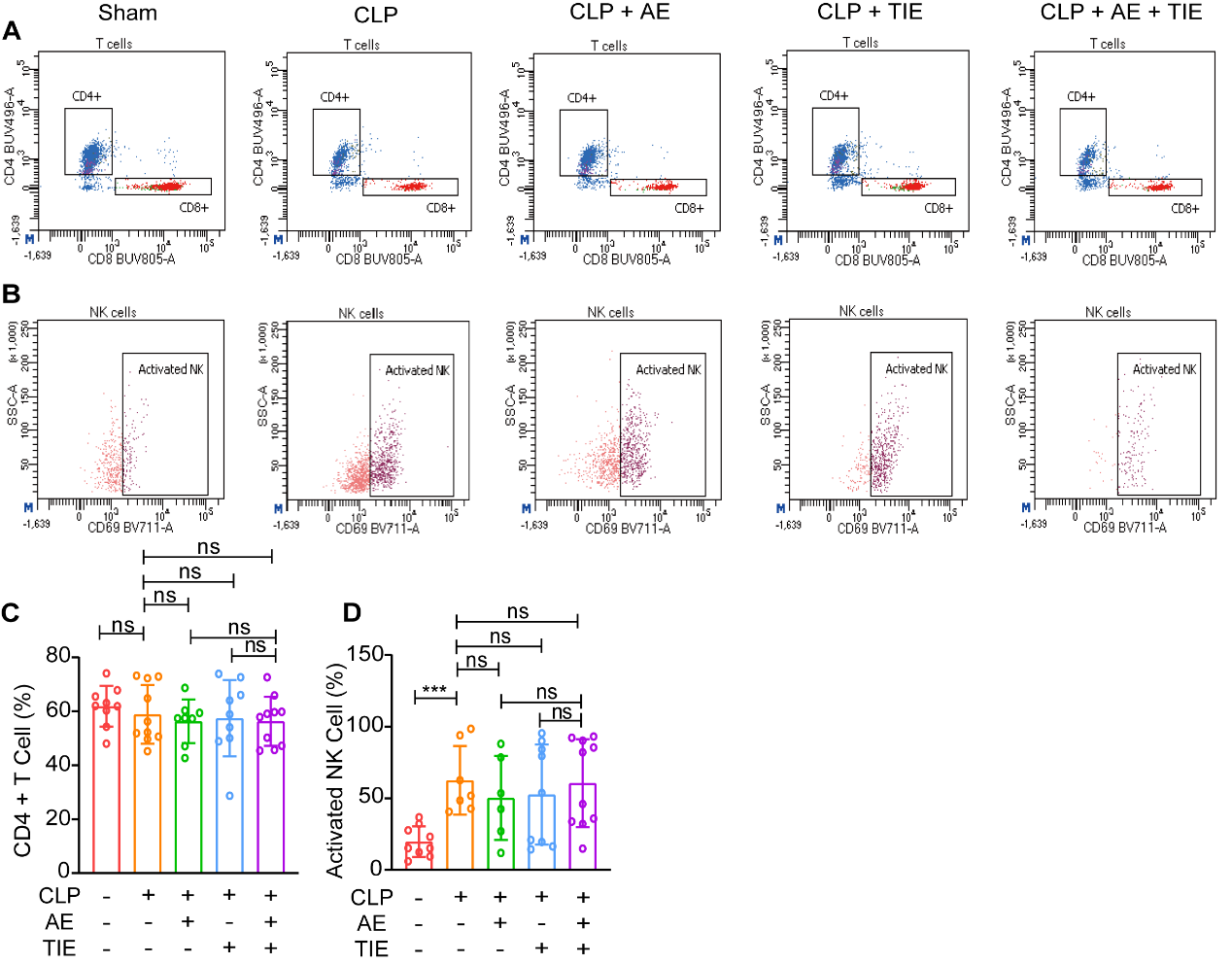


**Fig. S2. Impact of AE and TIE treatment on peripheral blood immune cells in mouse model of sepsis.** (A) CD4^+^ T cells gating plots; (B) Activated NK cells gating plots. Quantitative analysis of (C) CD4^+^ T cells, and (D) activated NK cells. Statistical analysis was performed using one-way ANOVA followed by Tukey’s *post hoc* test (*n* = 5-10). *** *P* < 0.001; ns, not significant.


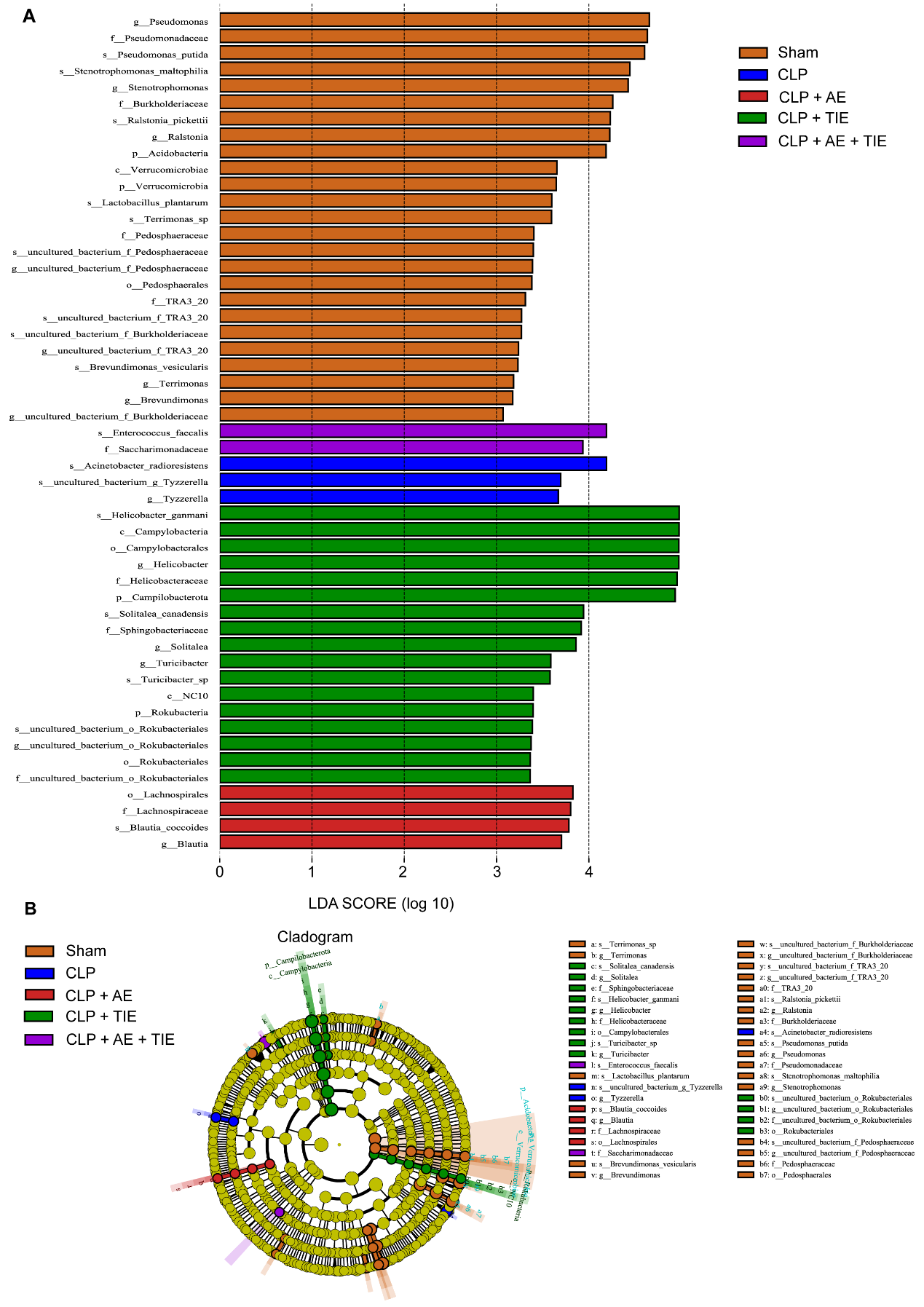


**Fig. S3. Linear discriminant analysis effect size** **(LEfSe) analysis of differences in peritoneal flora in mice.** (A) LEfSe comparison of the gut microbiota (LDA > 4.0); (B) Cladogram generated from the LEfSe analysis.


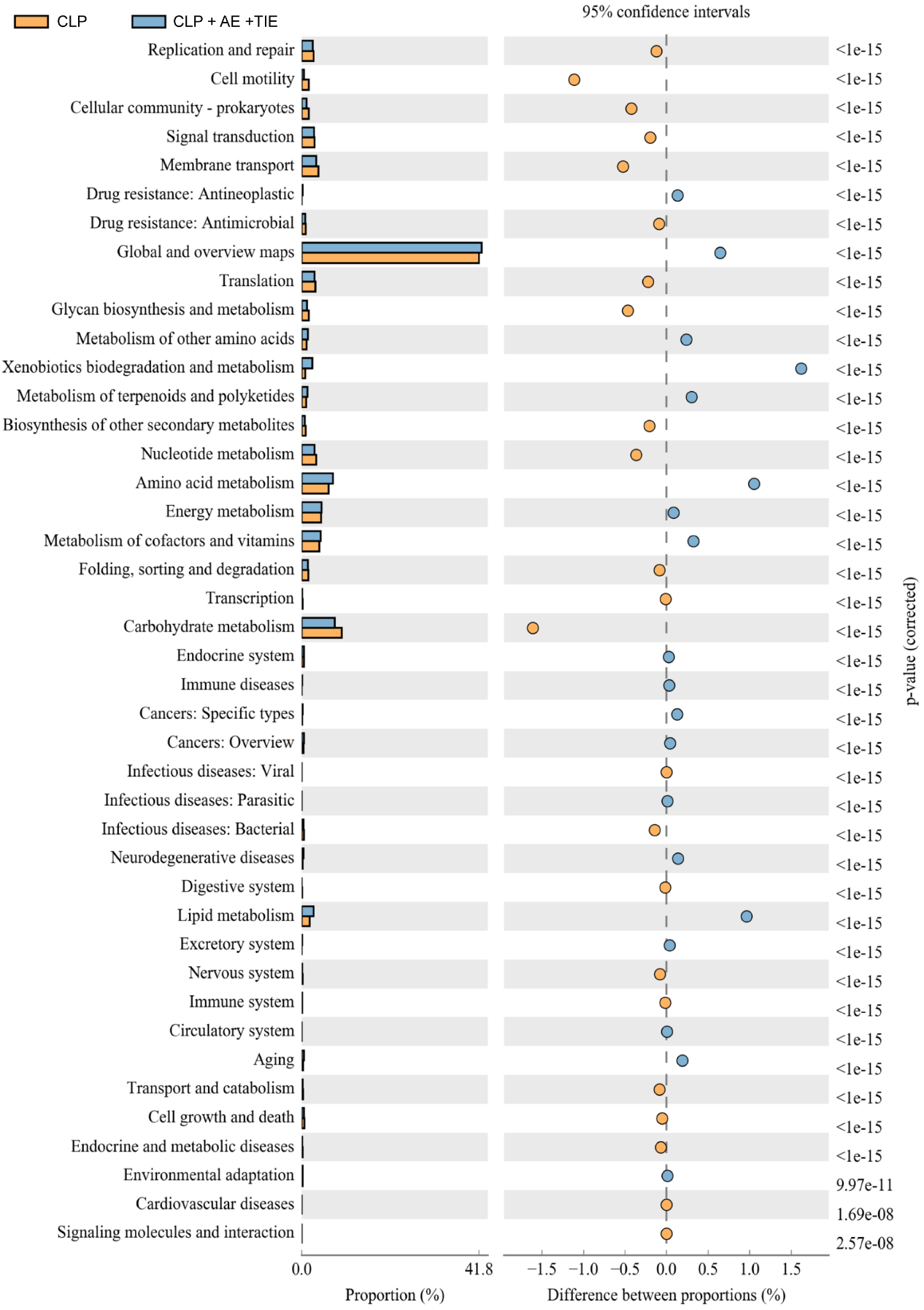


**Fig. S4. Comparative metagenomic prediction of intestinal microbial functions between CLP and CLP+AE+TIE treated wild-type mice using communities by** **reconstruction of unobserved states (PICRUSt2) tool**. This analysis explores the predicted alterations in the functional profile of intestinal microbiota between mice subjected to CLP alone and those treated with CLP in conjunction with AE and TIE, leveraging the PICRUSt2 to gain insights into microbial functional capabilities.


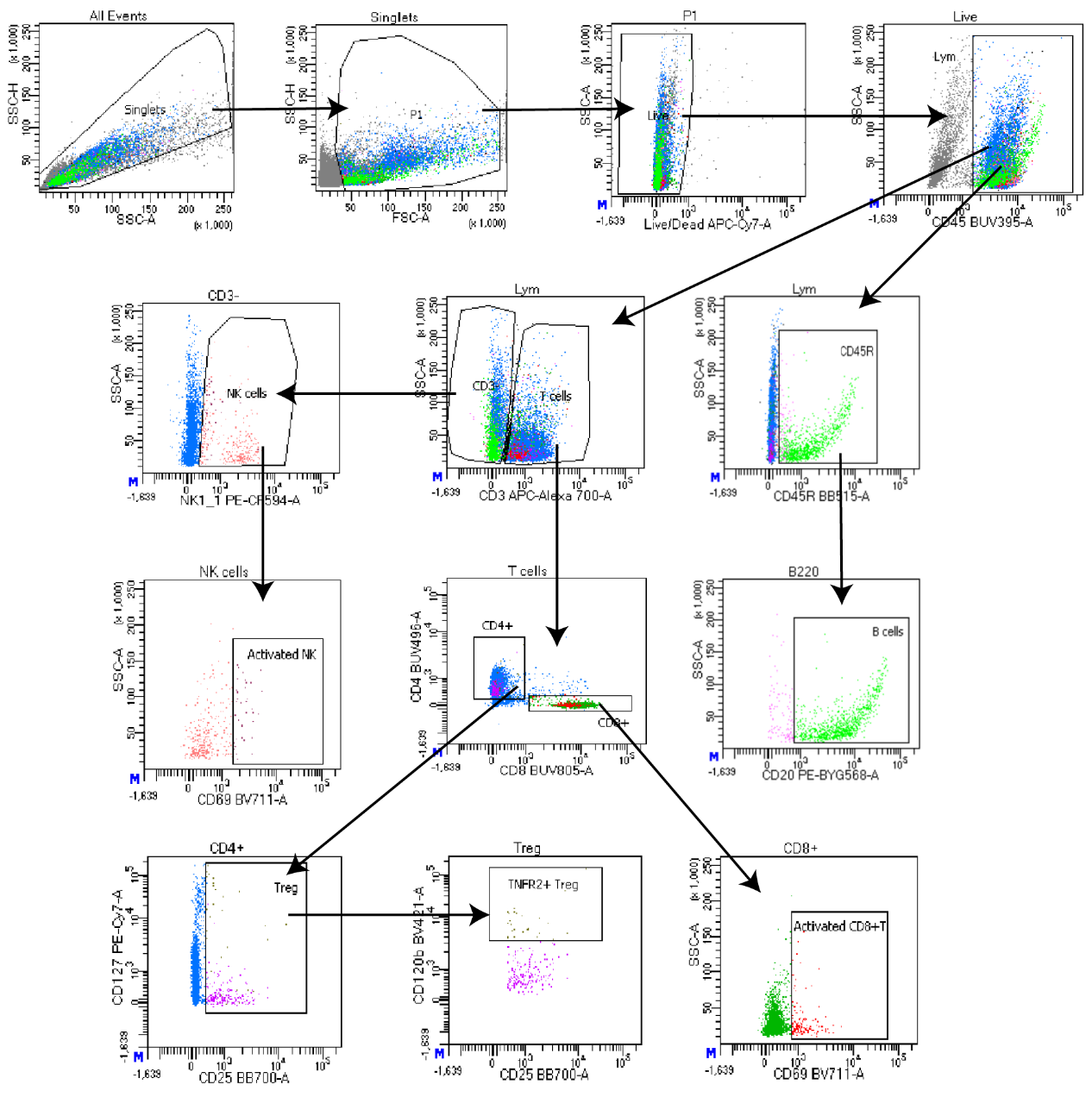


**Fig. S5. Gating strategies for flow cytometry.**

**
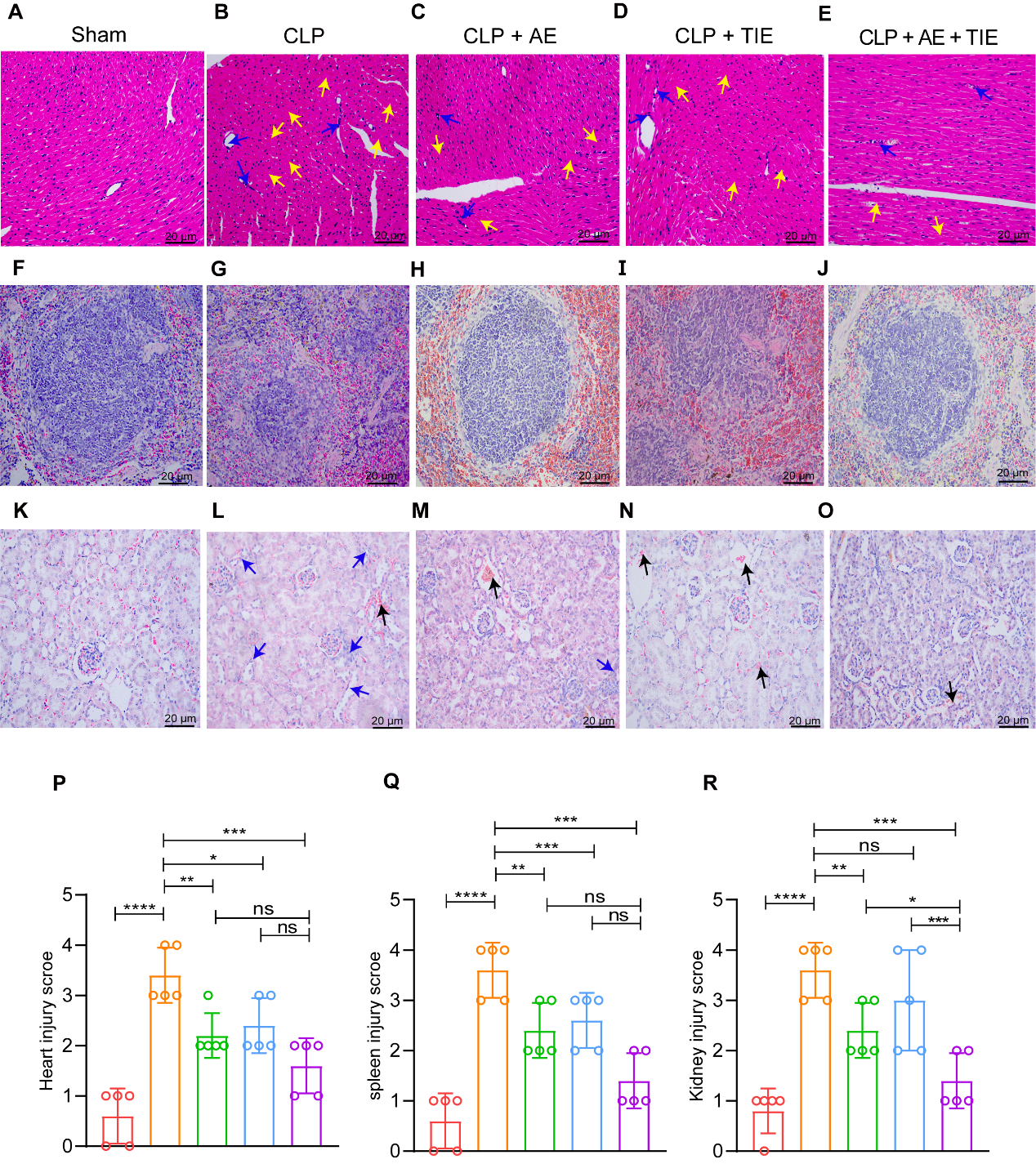
**

**Fig. S6. AE + TIE effects on Heart and spleen in a CLP-induced sepsis mouse model.** Microscopic images of heart (A-E), spleen (F-J) and kidney (K-O) lesions stained with hematoxylin and eosin (H&E), observed at 200× magnification. Indicators of tissue damage include infiltration by inflammatory cells (blue arrows), thickening of alveolar walls (black arrows), and cardiomyocyte necrosis (yellow arrows). Pathological scoring for heart (P), spleen (Q) and kidney (R) tissues. Data are depicted as mean ± SEM. Statistical analysis was conducted using Tukey's *post hoc* test following one-way ANOVA (*n* = 5). * *P* < 0.05, ** *P* < 0.01, *** *P* < 0.001, and **** *P* < 0.0001; ns, not significant. Scale bars: 20 μm.
